# Food Noise Among Healthcare Students and Healthcare Professionals in Al-Ahsa, Saudi Arabia: A Cross-Sectional Study

**DOI:** 10.3390/nu18172824

**Published:** 2026-08-28

**Authors:** Saba Alarfaj, Manal Alqahtani, Abdullah Almaqhawi

**Affiliations:** 1 Clinical Pharmacy, College of Clinical Pharmacy, King Faisal University, Al Ahsa P.O. Box 400, Saudi Arabia; 222405835@student.kfu.edu.sa (S.A.); 222441140@student.kfu.edu.sa (M.A.); 2 Department of Family and Community Medicine, College of Medicine, King Faisal University, Al Ahsa P.O. Box 400, Saudi Arabia

**Keywords:** food noise, food cravings, eating disorders, perceived stress, healthcare students, Saudi Arabia

## Abstract

Background: Food noise describes persistent, intrusive thoughts about food, eating, and cravings that occur in the absence of physiological hunger. Its occurrence among healthcare students and professionals in Saudi Arabia is unknown. This study measured food noise in this group in Al-Ahsa, described the proportion meeting study-defined severity thresholds, and identified its main correlates. Methods: In this cross-sectional study, healthcare students and professionals in Al-Ahsa completed a bilingual (Arabic–English) electronic questionnaire capturing demographic and lifestyle characteristics, a 14-item food noise scale adapted from the Food Thought Suppression Inventory (each item rated 1–5; total range 14–70), the SCOFF eating disorder screen, and the four-item Perceived Stress Scale. The factor structure of the food noise scale was examined by exploratory factor analysis with parallel analysis and random split-half replication. Severity was graded from the mean item score as low, moderate, or high using exploratory thresholds, and associations were tested using t-tests, one-way analysis of variance, the Pearson correlation, and complete-case multivariable linear regression with robust standard errors and six sensitivity analyses. Results: Among 593 participants (mean age 25.7 years; 67.5% women; 60.0% students), the food noise scale was unidimensional (Kaiser–Meyer–Olkin 0.95; one eigenvalue above both unity and the parallel analysis threshold; 41.8% of variance explained; loadings 0.50 to 0.74) and internally consistent (Cronbach alpha 0.91). The mean total score was 41.9 (standard deviation 12.7). At least moderate food noise was present in 75.9% of participants (95% CI 72.3 to 79.2) and high food noise in 29.0% (95% CI 25.5 to 32.8). A positive SCOFF screen was recorded in 70.2% (95% CI 66.3 to 73.7), and the mean perceived stress score was 8.1 (standard deviation 2.8). In the adjusted model (*n* = 573; unadjusted R-squared 0.279, adjusted R-squared 0.266), food noise was independently associated with the SCOFF score (B 3.85 per point) and perceived stress (B 0.69 per point), both of which held across every sensitivity analysis, although both relationships proved concave rather than linear. Weaker associations with age (B 0.19 per year) and any night shift work (B 2.45) did not survive alternative model specifications, and crude differences by sex, role, weekly hours, and fasting did not persist after adjustment. Conclusions: Food noise was common in this sample and tracked eating disorder risk and perceived stress more closely than demographic or occupational characteristics. Because recruitment was by convenience and the severity thresholds are exploratory rather than validated, the proportions reported here describe this sample and should not be read as population prevalence. The findings provide a first benchmark for the construct in this population and support attention to the cognitive dimension of eating within health professions settings.

## 1. Introduction

Food noise refers to persistent, intrusive thoughts about food, eating, and cravings that surface in the absence of physiological hunger and pull at attention during ordinary daily activities [1]. The term began as informal shorthand and moved into clinical and public conversation once patients taking glucagon-like peptide-1 receptor agonists described these thoughts growing quieter as their appetite and body weight fell [2,3]. Conceptually, it sits close to food cue reactivity: the cluster of cognitive, affective, and physiological responses triggered by the sight, smell, or mental image of palatable food [1,4]. Repeated exposure to energy-dense foods strengthens the reward value of food-associated cues while weakening inhibitory control, so that a cue alone can prompt craving and food seeking through mesocorticolimbic reward circuitry [4,5].

Why do these thoughts persist? The elaborated intrusion theory of desire offers one explanation, framing craving as an initially automatic intrusion that is then elaborated in working memory, often as vivid imagery, and grows harder to dismiss the more attention it draws [6,7]. Experimental work backs this up: craving-related thoughts subside when the elaboration is interrupted, which supports the view that food noise is a cognitively maintained experience rather than a simple response to hunger [6]. The same logic helps explain why appetite-blunting agents are reported to dull the intrusive quality of food thoughts, and why interest in measuring food noise has grown [2,3].

Healthcare students and professionals are a group in whom these mechanisms may be amplified. Training and clinical work bring high levels of stress and burnout: pooled estimates put stress at around forty per cent among medical students, and burnout has been documented across physician populations worldwide [8,9]. Stress, in turn, shapes eating. It activates the hypothalamic–pituitary–adrenal axis and the reward system and can heighten the motivational pull of calorie-dense food, though whether intake actually rises varies from person to person and setting to setting [10,11]. Irregular schedules add to the problem, since insufficient sleep and circadian misalignment raise appetite by tilting the balance of ghrelin and leptin and by increasing energy intake—effects quantified in controlled studies and reviews [12,13,14]. Night shift work, common in nursing and other clinical roles, has in turn been linked prospectively to weight gain and to daytime sleepiness among hospital staff [15,16].

Disordered eating is also relatively common here, and food noise plausibly overlaps with it. Eating disorder symptoms affect a meaningful minority of medical students, and concerns about diet and body image are widespread in this group [8,17]. Brief screening tools such as the SCOFF questionnaire are widely used to flag probable eating disorders in student and community samples, though they are prone to over-identification once applied outside clinical settings [18,19,20]. Perceived stress, for its part, is usually captured with the Perceived Stress Scale, whose short forms have been tested across student and worker populations [21,22].

In Saudi Arabia, obesity and unfavourable dietary patterns are prominent public health concerns, and national surveys have documented heavy consumption of sugar-sweetened and energy drinks alongside frequent weight-related behaviours among adults [23,24]. Disordered eating has likewise been reported in Saudi university students with standardised tools, with a substantial share of them exceeding clinical risk thresholds [25]. Food noise, however, has not been studied in healthcare students and professionals in the Kingdom, and no local data exist for Al-Ahsa. We therefore set out to measure food noise in this population and to describe the proportion of participants meeting a study-defined threshold for it, to map its distribution across demographic and lifestyle characteristics, to compare students with professionals, and to identify the factors most strongly associated with it. Because the sample was recruited by convenience and the thresholds were defined from the response scale rather than from any clinical criterion, the study was not designed to estimate population prevalence.

## 2. Materials and Methods

### 2.1. Study Design and Setting

A cross-sectional, questionnaire-based study was conducted among healthcare students and healthcare professionals in Al-Ahsa, Saudi Arabia, and was reported in accordance with the STROBE statement for cross-sectional studies [26] (Appendix A). The study population was drawn from universities, hospitals, and primary healthcare centres in the governorate. Recruitment ran from November 2025 to May 2026. Four hospitals and the health-related colleges of King Faisal University took part, and the questionnaire was additionally circulated through social media platforms (including WhatsApp and X/Twitter) and the Barcoda survey-distribution service, so an unknown share of respondents was reached outside the participating institutions. Data were collected through a self-administered electronic questionnaire, available in Arabic and English so that respondents could complete it in their preferred language. Because the questionnaire was circulated as an open link rather than sent to an enumerated list of invitees, the number of people who saw the invitation is not recorded, and a response rate cannot be calculated. Nor can a reliable denominator be given for the eligible population, which spans several institutions and several professional cadres across the governorate and is not enumerated in any single register; the figures reported here are therefore proportions within the achieved sample and carry no implied sampling fraction. The survey platform was set to accept one response per account. Item completion was near-complete: twelve respondents left the night shift item blank, which was phrased “if any”, and one left the physical activity item blank; every other item was answered by all 593 participants included in the analysis.

### 2.2. Participants and Eligibility

Eligible participants were adults aged 18 years or older who were either healthcare students or practising healthcare professionals and who were resident in Al-Ahsa. Participation required electronic informed consent. Respondents who declined consent, who did not belong to a healthcare field, or who were younger than 18 years were not included in the analysis. Records with an implausible or non-interpretable age entry and records missing any item of the food noise scale were also excluded. An age entry was treated as implausible when the recovered value fell outside 18 to 80 years and as non-interpretable when it contained no recoverable number; entries written in words or with a unit attached, such as “22 years” or the Arabic for “twenty-five”, were converted to numbers and retained. All records were screened for duplicate and automated submissions by checking for identical response patterns across the full set of demographic, lifestyle, and scale items. Sampling followed a convenience approach because of the electronic distribution method.

### 2.3. Questionnaire and Measures

The questionnaire comprised four parts. The first part collected demographic and lifestyle information, including age, sex, professional role (student or professional), healthcare discipline, average weekly working or study hours, frequency of physical activity, the number of night shifts performed per month, and whether the respondent fasted regularly for religious or health reasons. No anthropometric measurements were available for analysis: the analysis dataset contains no height or weight values, so body mass index could not be derived and could not be entered into the descriptive table, the adjusted model, or a sensitivity analysis. The consequences of this are set out in limitations.

The second part measured food noise using a 14-item battery that captured intrusive, persistent, and difficult-to-control thoughts about food, in line with the conceptual framing of food cue reactivity described by Hayashi and colleagues [1]. Each item was rated on a five-point Likert scale ranging from 1 (strongly disagree) to 5 (strongly agree), with higher scores reflecting greater food noise. A total score was obtained by summing the 14 items (possible range of 14 to 70), and a mean item score (range 1 to 5) was derived for interpretation against the scale anchors. The 14 items were adapted from the Food Thought Suppression Inventory [27], which assesses intrusive and hard-to-control thoughts about food, and were framed in line with the food cue reactivity model. The original Food Thought Suppression Inventory comprises 15 items. One item (“I have thoughts about food I cannot stop”) was judged to overlap conceptually with a retained item (“I think about food constantly”) and was omitted when the questionnaire was built, so the instrument administered to participants contained 14 items, and no item was removed after data collection. Because the omission preceded data collection, a sensitivity analysis retaining the full 15-item version is not possible in this dataset. The Arabic version of the 14 items was produced by forward translation, independent back translation, reconciliation by an expert panel.

The third part screened for disordered eating using the SCOFF questionnaire [18], a five-item instrument with yes or no responses. One point was assigned for each affirmative answer, giving a total of 0 to 5, and a score of 2 or higher was taken to indicate a probable eating disorder, consistent with the original threshold. The validated Arabic version of the SCOFF [28] was used for Arabic-speaking respondents.

The fourth part assessed perceived stress with the four-item version of the Perceived Stress Scale [21], using its validated Arabic version for Arabic-speaking respondents [29] Items were scored from 0 to 4, the two positively worded items were reverse-scored, and a total score from 0 to 16 was computed, with higher values indicating greater perceived stress. A single additional question, based on the Standardised Nordic Musculoskeletal Questionnaire [30] and its validated Arabic adaptation [31] asked respondents to indicate the site of any musculoskeletal pain or discomfort experienced during the preceding 12 months that had interfered with work or daily activities. The response options were neck, shoulders, lower back, knees, and no pain or discomfort, and a single option was selected.

### 2.4. Operational Definitions of Food Noise

Because no validated diagnostic cut-point for food noise is currently established, severity was defined operationally from the mean item score using the mid-points between adjacent Likert anchors. A mean below 2.5 was classified as low food noise, a mean from 2.5 to below 3.5 as moderate food noise, and a mean of 3.5 or above as high food noise. These thresholds are descriptive and were specified in advance of the analysis, but they are arbitrary in the sense that they derive from the response scale rather than from any external criterion, and they have not been calibrated against a clinical or diagnostic standard. They therefore define a study-specific operational category and not a clinical state. Throughout this report the resulting figures are described as the proportion of participants meeting the study-defined threshold, and because the sample was recruited by convenience, they are not offered as population prevalence estimates and are not comparable with figures generated under different thresholds.

### 2.5. Statistical Analysis

Data were cleaned and analysed in Python (version 3.14.2) (pandas, SciPy, statsmodels, and factor_analyzer); equivalent procedures are available in SPSS (version 31.0). Free-text role entries were mapped to a healthcare discipline and to a student or professional category using a predefined classification scheme. Continuous variables were summarised as means with standard deviations and, because several distributions were skewed, also as medians with interquartile ranges; categorical variables were summarised as frequencies with percentages, and proportions were reported with Wilson 95% confidence intervals. Two categorical predictors were collapsed before the multivariable model was fitted. Physical activity was dichotomised as regular against irregular or none, and night shift frequency as any against none. We should be explicit that neither decision was prespecified in the protocol: both were taken after inspecting the bivariate results because the irregular and inactive groups did not differ on the outcome and because the three night shift categories differed from the no-shift group but not from one another. These are therefore data-informed, exploratory coding choices rather than prespecified ones, and both are reported alongside a sensitivity analysis retaining the original categories. Weekly hours were kept in their three original categories throughout, and the four-level night shift variable was retained for the bivariate analysis so that the shape of the association remains visible. Internal consistency of the food noise scale was assessed with the Cronbach alpha coefficient and item-rest correlations. To examine dimensionality, the suitability of the data for factor analysis was first assessed with the Kaiser–Meyer–Olkin (KMO) measure of sampling adequacy and Bartlett’s test of sphericity. Exploratory factor analysis was then performed using principal-axis factoring, with the number of factors determined by parallel analysis against the 95th percentile of eigenvalues from 500 random datasets of the same size, the scree plot, and eigenvalues greater than one; an oblique (promax) rotation was applied to allow for correlated factors. Items with a loading of 0.40 or above were regarded as salient, and the proportion of variance explained was reported. Because the same sample was used to adapt the scale and to estimate its structure, the solution was cross-checked in a random split-half of the data, and the two sets of loadings were compared with Tucker’s coefficient of congruence. All psychometric findings are reported as exploratory.

Associations between the food noise total score and participant characteristics were examined with the independent-samples *t*-test for two-group comparisons and one-way analysis of variance for comparisons across more than two groups, with the Cohen d and eta-squared reported as effect sizes. Relationships with continuous variables (age, perceived stress, and the SCOFF total) were assessed with the Pearson correlation coefficient. The association between food noise severity category and role group was tested with the chi-square test. These bivariate comparisons were exploratory and were not adjusted for one another, so the Benjamini–Hochberg false discovery rate was applied across the ten tests and q values are given alongside the nominal *p* values. A multivariable linear regression model was then fitted with the food noise total score as the dependent variable and age, sex, role group, physical activity, night shift work, weekly hours, regular fasting, perceived stress and the SCOFF score as independent variables. Predictors were chosen before analysis on substantive grounds rather than by stepwise selection: age, sex, and role group as demographic characteristics; physical activity, night shift work, weekly hours, and fasting as the lifestyle exposures the study was designed to test; and perceived stress and eating disorder risk as the two psychological constructs named in the objectives. All were entered together and retained regardless of significance. Unstandardised coefficients with 95% confidence intervals, standardised coefficients, and the model coefficient of determination were reported, and crude coefficients from single-predictor models are presented alongside the adjusted ones.

The primary model used complete-case analysis; no values were imputed, and no missing category was created; and the number of observations contributing to each predictor and to the model is reported. Model assumptions were checked by inspection of residual skewness and kurtosis with the Shapiro–Wilk test, the Breusch–Pagan test for heteroscedasticity, the Ramsey RESET test for functional form, Cook’s distance for influential observations, and the variance inflation factor (VIF) for multicollinearity. Heteroscedasticity-consistent (HC3) standard errors were computed for every coefficient and are reported alongside the conventional ones.

Six sensitivity analyses were run. The first four were prespecified: refitting the model on all 593 participants with a missing category indicator for the lifestyle variables; refitting after excluding observations with a Cook’s distance above 4/*n*; recomputing the correlation and the adjusted association between food noise and the SCOFF screen after removing the conceptually overlapping items from each instrument, together with item-level correlations between SCOFF item 5 and each food noise item; and repeating the analyses after excluding respondents who gave the same answer to all 14 food noise items. Two further analyses were added in revision. The fifth refitted the model with physical activity and night shift frequency in their original categorical form, with joint Wald tests of each set of indicator variables and a test of whether the three night shift categories differ from one another. The sixth relaxed the assumption of linearity for the three continuous predictors, first by adding quadratic terms for age, perceived stress and the SCOFF score and then by fitting restricted cubic splines with three knots placed at the 10th, 50th and 90th centiles of each; the added terms were tested jointly and individually, and the Ramsey RESET test was repeated on each refitted model. A two-sided *p* value below 0.05 was considered statistically significant.

The sample size was determined by the number of eligible responses received within the recruitment window rather than by an a priori power calculation, and no target was set in advance. Rather than compute retrospective power, which adds little once effect estimates and confidence intervals are available, we report the precision the achieved sample delivers for each of the three objectives. For estimating proportions, 593 participants give a 95% confidence interval no wider than about 4.0 percentage points on either side of a proportion near 50%, and 3.4 percentage points around the observed figure of 75.9%. For the factor analysis, the subject-to-item ratio is 42:1, well above the commonly cited minimum of 10:1. For the regression, 573 complete cases with ten predictors correspond to a smallest detectable partial R-squared of about 0.014 for a single predictor at a two-sided alpha of 0.05, which is a small effect. The confidence intervals should be read as the primary guide to the precision of each estimate.

### 2.6. Ethical Considerations

The study was conducted in accordance with the principles of the Declaration of Helsinki. Participation was voluntary and anonymous, electronic informed consent was obtained before the questionnaire could be accessed, and no personally identifying information was collected. Ethical approval was obtained from the relevant institutional review board.

## 3. Results

### 3.1. Participant Flow and Sample Characteristics

A total of 644 responses were received. Seven respondents who declined consent were excluded, followed by 38 respondents who did not belong to a healthcare field, a group that included the five records containing no answers beyond the consent question and the respondents aged under 18, and six respondents with an implausible or non-interpretable age (two entries of 100 or more, one four-digit entry, one entry that was a year of birth, and two free-text answers containing no number). No eligible respondent had missing food noise items. The final analytic sample comprised 593 participants. The duplicate screen identified one pair of consecutive records that was identical on every demographic, lifestyle, and scale variable and is therefore likely to represent a single respondent submitting twice; the pair was retained in the primary analysis because it could not be verified as a duplicate, and removing one of the two records changed no estimate in this report. Thirty-one participants (5.2%) gave the same response to all 14 food noise items.

The mean age was 25.7 years (standard deviation 6.6; median 23, interquartile range 22 to 27; range 18 to 59). Women accounted for 67.5% of the sample (400 of 593). Students made up 60% of participants and professionals 40%. Nursing, pharmacy, and medicine were the most common disciplines. The full sample characteristics are presented in Table 1. Students and professionals differed on the characteristics that follow from the roles themselves: professionals were older (30.3 years, standard deviation 8.2, against 22.7, standard deviation 2.5; *p* < 0.001), a larger share of them were men (41.2% against 26.8%; *p* < 0.001), they worked longer weeks (38.7% above 40 h against 24.2%; *p* < 0.001), and they worked more night shifts (75.2% any night shifts against 33.7%; *p* < 0.001). The two groups did not differ in physical activity (*p* = 0.52), regular fasting (*p* = 0.43), the SCOFF total (*p* = 0.95), or perceived stress (*p* = 0.13).

### 3.2. Prevalence and Severity of Food Noise

The food noise scale showed high internal consistency (Cronbach alpha 0.909), and all 14 items correlated positively with the remainder of the scale, with item-rest correlations ranging from 0.47 to 0.70, supporting a single summed score. The mean total food noise score was 41.9 (standard deviation 12.7; median 43, interquartile range 35 to 50; observed range 14 to 70), corresponding to a mean item score of 2.99 on the five-point scale.

Before scoring, the suitability of the food noise items for factor analysis was confirmed by the Kaiser–Meyer–Olkin measure of 0.95 for the scale as a whole, with item-level values from 0.92 to 0.97, and by a significant Bartlett’s test of sphericity (χ^2^ = 3301.9, *df* = 91, *p* < 0.001). Exploratory factor analysis using principal-axis factoring with parallel analysis indicated a one-factor solution. Only the first eigenvalue exceeded unity (6.42, against 0.92 for the second and 0.85 for the third), and only the first exceeded the 95th percentile of eigenvalues generated from random data of the same size (1.32 for the first factor and 1.25 for the second); the scree plot showed a single dominant component (Supplementary Figure S1). The one-factor solution accounted for 41.8% of the total variance. All items loaded saliently (≥0.40) on the single factor, with loadings ranging from 0.50 to 0.74 and communalities from 0.25 to 0.54, supporting the use of a unidimensional total score. The full factor loadings are presented alongside the item descriptive statistics in Table 2. A two-factor promax solution was examined for completeness and was not retained: the second factor accounted for only a further 8.9% of variance, the two factors correlated at 0.72, and the second factor was defined by a single item with a loading of 0.96 and a communality of 0.95, a pattern that indicates an unstable rather than a substantive dimension. Because the same sample was used to adapt the scale and to estimate its structure, the one-factor solution was re-estimated in two random halves of the data (*n* = 296 and *n* = 297); it replicated closely, with variance explained of 41.2% and 42.4%, Cronbach alpha of 0.906 and 0.910, and a Tucker congruence coefficient of 0.998 between the two sets of loadings. These psychometric results are exploratory, and confirmation in an independent sample is still required.

**Figure 1:**
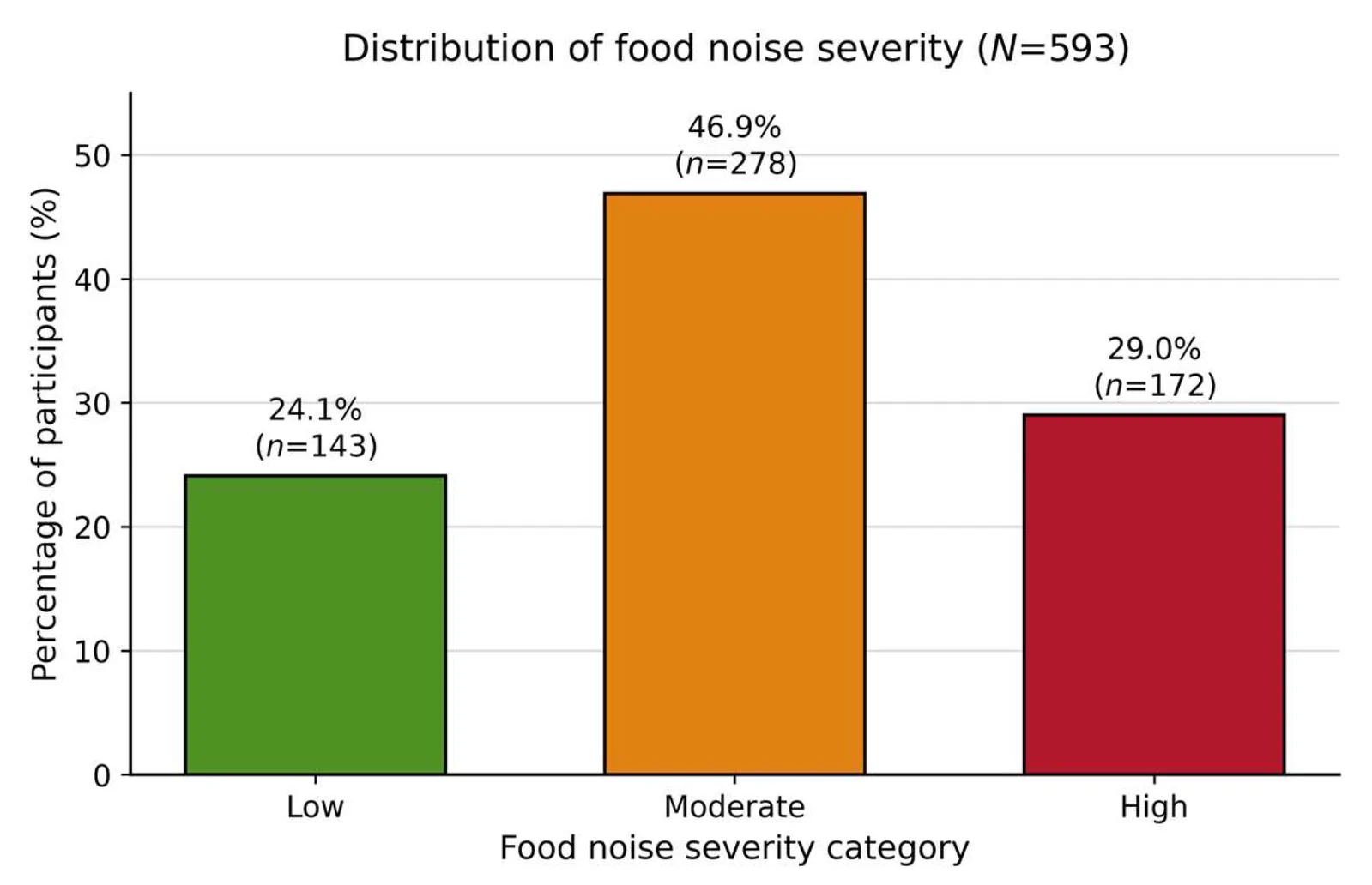
Distribution of food noise severity categories in the study sample.

Using the predefined thresholds, 24.1% of participants (*n* = 143; 95% CI 20.8 to 27.7) reported low food noise, 46.9% (*n* = 278; 95% CI 42.9 to 50.9) moderate food noise, and 29.0% (*n* = 172; 95% CI 25.5 to 32.8) high food noise. Food noise of at least moderate severity was therefore present in 75.9% of the sample (*n* = 450; 95% CI 72.3 to 79.2), while 29.0% reached the high-severity band. These figures describe the proportion of this convenience sample meeting the study-defined thresholds rather than a population prevalence. The distribution of severity categories is shown in Figure 1.

**Figure 2:**
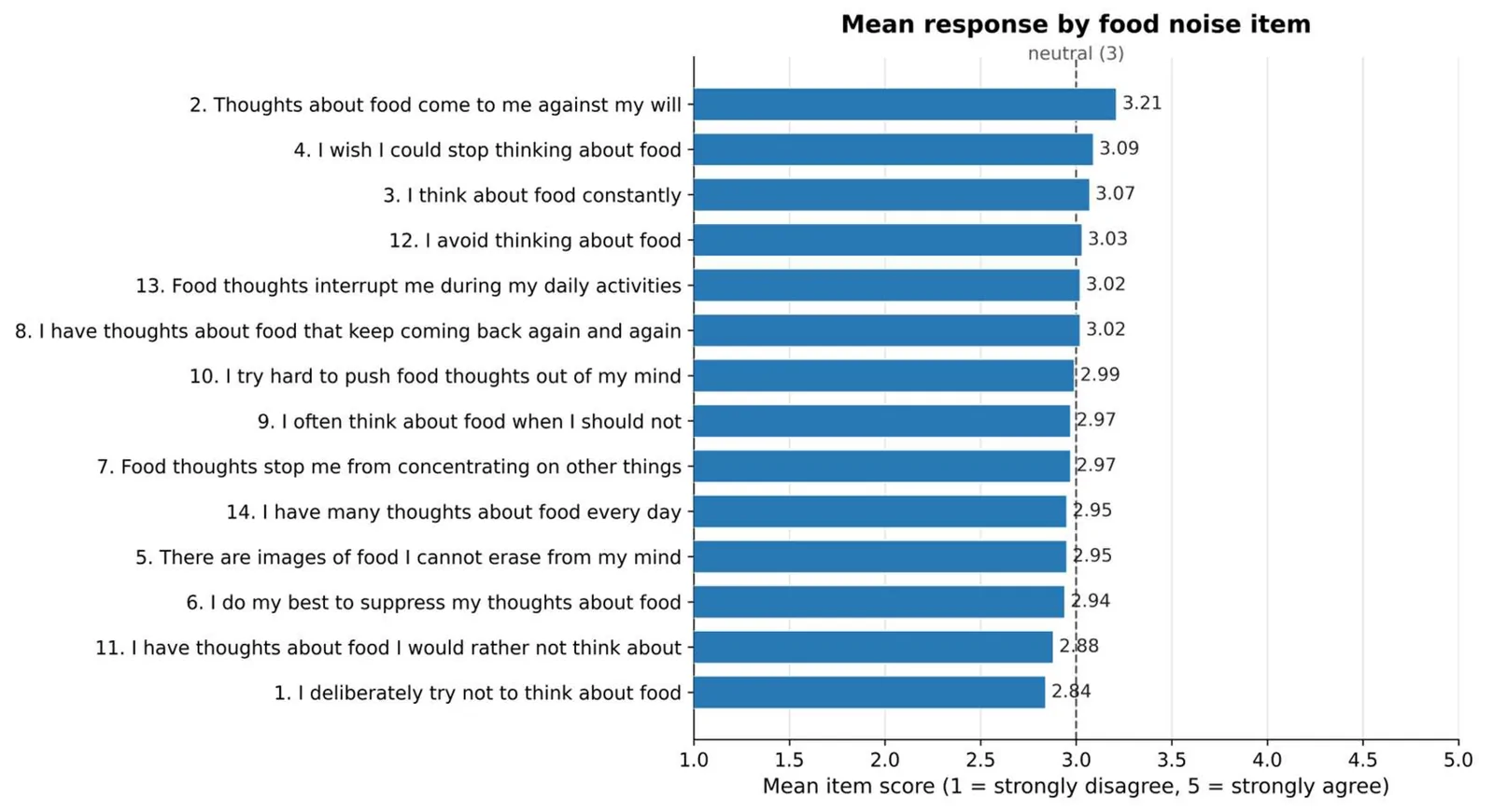
Mean response for each food noise item, ordered from lowest to highest. The dashed line marks the neutral mid-point.

At the item level, the highest-rated experiences concerned involuntary and persistent thoughts about food, whereas items describing deliberate avoidance or suppression of food thoughts received slightly lower ratings. The mean item scores clustered around the neutral mid-point, as detailed in Table 2 and illustrated in Figure 2.

### 3.3. Disordered Eating, Perceived Stress, and Musculoskeletal Symptoms

On the SCOFF screen, the mean score was 2.16 (standard deviation 1.4; median 2, interquartile range 1 to 3), and 70.2% of participants (*n* = 416; 95% CI 66.3 to 73.7) screened positive for a probable eating disorder using the threshold of two or more affirmative answers. Endorsement was high on every item and ranged from 34.2% to 49.1%, including 43.2% for the item asking about deliberate vomiting when uncomfortably full, a figure far above what is usually reported in non-clinical samples and one that bears on the interpretation of the overall screen result (see Section 4). Item-level endorsement is shown in Table 3. The mean perceived stress score (PSS-4) was 8.1 (standard deviation 2.8; median 8, interquartile range 6 to 10) on the 0 to 16 range.

Musculoskeletal pain or discomfort during the preceding 12 months that had interfered with work or daily activities was reported by 491 participants (82.8%). The lower back was the most frequently reported site (166, 28.0%; 95% CI 24.5 to 31.7), followed by the shoulders (147, 24.8%; 95% CI 21.5 to 28.4), the neck (107, 18.0%; 95% CI 15.2 to 21.3), and the knees (71, 12.0%; 95% CI 9.6 to 14.8); 102 participants (17.2%) reported no pain or discomfort. The food noise total score did not differ between participants reporting any musculoskeletal symptom and those reporting none (42.1, standard deviation 12.6, against 41.4, standard deviation 12.9; t = 0.55, *p* = 0.58; d = 0.06), and the variable was therefore not carried into the multivariable model.

### 3.4. Factors Associated with Food Noise

**Figure 3:**
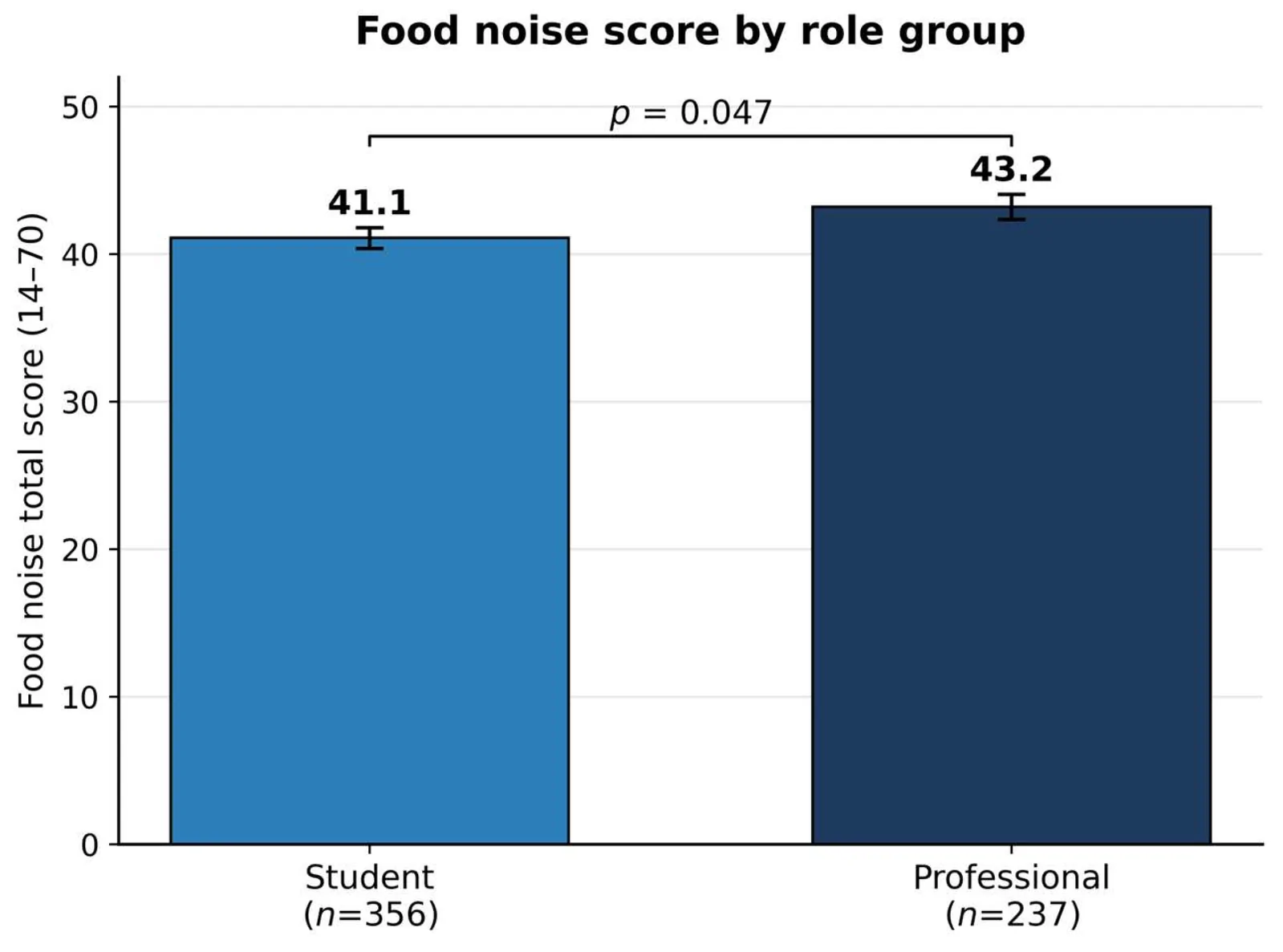
Mean food noise total score by role group. The error bars show the standard error of the mean.

**Figure 4:**
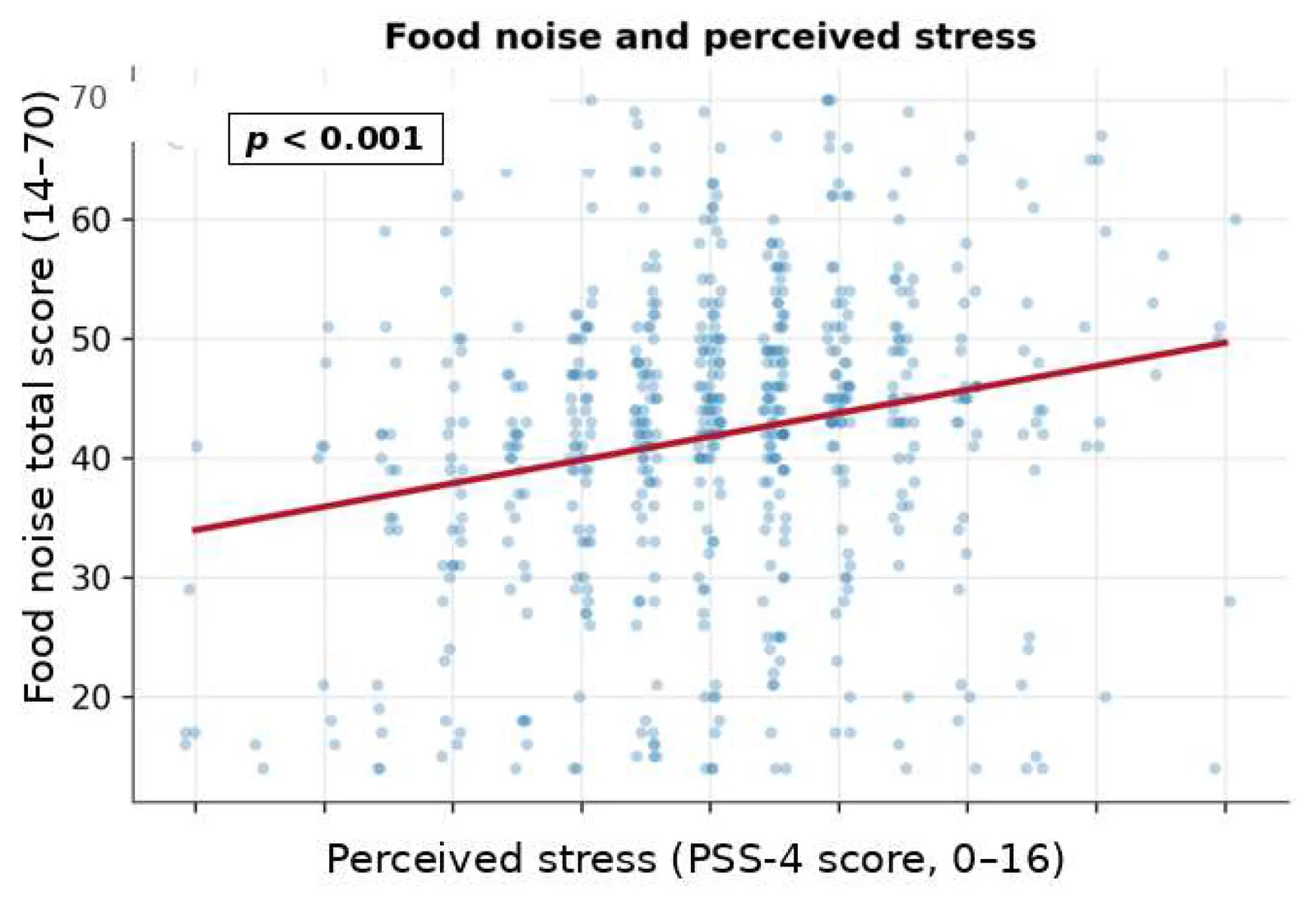
Relationship between perceived stress (PSS-4) and the food noise total score, with fitted regression line.

**Figure 5:**
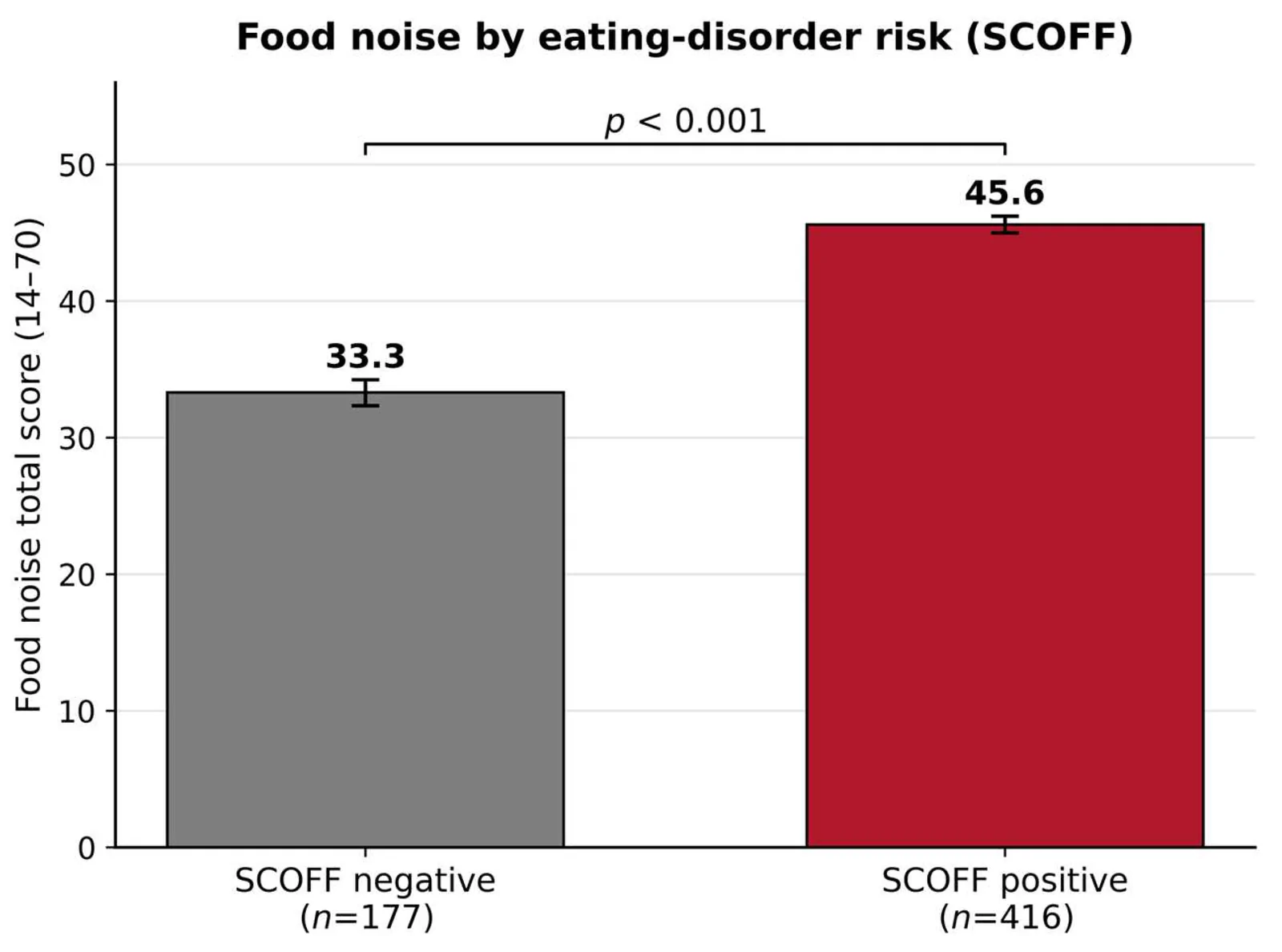
Mean food noise total score by SCOFF screen result. The error bars show the standard error of the mean.

In unadjusted comparisons, the food noise total score was higher in men than in women and higher in professionals than in students, although both differences were small. Participants who fasted regularly scored higher than those who did not. The number of night shifts per month showed a threshold pattern rather than a gradient: participants working no night shifts scored lowest (38.7), while the three shift-exposed groups scored between 44.0 and 45.6 and differed little from one another, which is the basis for the any-against-none coding used in the adjusted model. Older age, higher perceived stress, and a higher SCOFF score were each associated with greater food noise, with the SCOFF score showing the strongest relationship. Weekly hours and physical activity were not significantly associated with food noise. After the Benjamini–Hochberg correction across the ten tests in Table 4, every result that was nominally significant remained so except the difference by role group (*p* = 0.047, q = 0.059). Food noise severity categories were not distributed differently across role groups (high severity in 31.1% of professionals against 27.8% of students; χ^2^ = 3.87, *df* = 2, *p* = 0.145), but it did differ by sex (χ^2^ = 8.66, *df* = 2, *p* = 0.013) and, most markedly, by the SCOFF screen result, with high severity in 36.9% of screen-positive against 11.3% of screen-negative participants (χ^2^ = 135.8, *df* = 2, *p* < 0.001). These bivariate results are summarised in Table 4, and the principal comparisons are displayed in Figure 3, Figure 4 and Figure 5.

### 3.5. Multivariable Analysis

The multivariable model was fitted by complete-case analysis. Of the 593 participants, 20 were excluded because of a missing or non-classifiable value on a lifestyle variable (18 night shifts, 1 physical activity, 1 weekly hours), leaving 573 observations; age, sex, role group, fasting, perceived stress, and the SCOFF score were complete for all 593. The model explained 27.9% of the variance in the food noise total score (unadjusted R-squared 0.279; adjusted R-squared 0.266; F *p* < 0.001; *n* = 573). After mutual adjustment, four variables remained independently associated with food noise: each additional point on the SCOFF score corresponded to an increase of 3.85 points in the food noise score, each additional point of perceived stress to an increase of 0.69 points, each additional year of age to an increase of 0.19 points, and any night shift work to an increase of 2.45 points. The first two of these held across every sensitivity analysis, and the last two did not, as set out in Section 3.6. The crude associations with sex, role group, weekly hours, and fasting were no longer significant once eating disorder risk and perceived stress were taken into account. Crude and adjusted coefficients are presented side by side in Table 5. Multicollinearity was not a concern: all variance inflation factors lay between 1.05 and 1.63, and perceived stress and the SCOFF score correlated only weakly with one another (r = 0.19). Residuals were close to symmetric (skewness −0.19, kurtosis 3.50) but departed slightly from normality (Shapiro–Wilk W = 0.993, *p* = 0.014), and there was evidence of heteroscedasticity (Breusch–Pagan LM = 61.4, *df* = 10, *p* < 0.001) and of departure from linearity in the fitted relationship (Ramsey RESET F = 12.56, *p* < 0.001), which is addressed by the non-linear models reported in Section 3.6. Heteroscedasticity-consistent (HC3) standard errors are shown in Table 5; they did not change any conclusion, and the age coefficient became more rather than less precise (*p* = 0.008). No observation was individually influential (maximum Cook’s distance 0.027; none above 1).

### 3.6. Sensitivity Analyses

The findings were stable across the four prespecified sensitivity analyses, which are summarised together with the two added in revision in Table 6. Retaining all 593 participants with a missing category indicator for the lifestyle variables reproduced the model closely (R-squared 0.284; SCOFF B 3.88, perceived stress B 0.64, age B 0.19, any night shifts B 2.37, *p* = 0.029). Excluding the 27 observations with a Cook’s distance above 4/*n* left the two principal coefficients unchanged in direction and larger in size (SCOFF B 4.16, perceived stress B 1.02), while the night shift coefficient fell to 1.51 and lost significance (*p* = 0.121). Excluding the 31 participants who gave identical answers to all 14 food noise items left the correlation between food noise and the SCOFF total unchanged at 0.48.

The overlap in item content between the two instruments was examined directly. Removing the two food noise items that most closely resemble SCOFF item 5 (“I think about food constantly” and “I have many thoughts about food every day”) did not weaken the correlation with the SCOFF total (r rose slightly from 0.477 to 0.491). Removing SCOFF item 5 itself reduced the correlation from 0.477 to 0.402, and removing the overlapping items from both instruments gave 0.415. At the item level, SCOFF item 5 correlated with the 14 food noise items in a narrow band from 0.19 to 0.35, and its correlations with the two items named above were among the lowest of the 14 (0.24 and 0.21) rather than the highest. In an adjusted model using the reduced 12-item food noise score as the outcome and the four-item SCOFF score as the predictor, the association remained strong and significant (B 3.42, 95% CI 2.66 to 4.18; β 0.35; *p* < 0.001). Content redundancy therefore accounts for part of the observed association but does not explain it.

Refitting the model with physical activity and night shift frequency in their original categorical form explained the same share of variance as the primary model (R-squared 0.279 in both) and fitted slightly less well once the extra parameters were counted (adjusted R-squared 0.263 against 0.266; Akaike information criterion 4381.0 against 4375.2). The coefficients for age, perceived stress and the SCOFF score were unchanged to two decimal places. The three night shift categories carried similar coefficients relative to no night shifts (one shift B 2.83, two shifts B 2.41, three or more B 2.16) and did not differ from one another (F = 0.08, *df* 2 and 559, *p* = 0.921), which supports collapsing them, but no single category reached significance (*p* = 0.062, 0.079 and 0.130 respectively), and the joint Wald test of the three was not significant (F = 1.70, *df* 3 and 559, *p* = 0.165). The significance of the night shift association is therefore dependent on the coding: the dichotomy concentrates the same effect into one parameter, and splitting it across three parameters removes it. The two physical activity categories behaved similarly and were jointly non-significant (F = 0.41, *df* 2 and 559, *p* = 0.665).

Relaxing the assumption of linearity changed the shape of two associations without changing their direction or significance. Adding quadratic terms for age, perceived stress and the SCOFF score improved the fit (R-squared 0.300 against 0.279; joint test of the three terms F = 5.62, *df* 3 and 559, *p* = 0.001), and the restricted cubic spline model performed similarly (R-squared 0.302; joint test F = 6.07, *p* = 0.001). The departure from linearity was carried by perceived stress (*p* = 0.002) and the SCOFF score (*p* = 0.010), not by age (*p* = 0.265). Both relationships were concave, so that each additional point added less than the one before: in the spline model the food noise score rose by 5.8 points between the SCOFF scores of 0 and 1 but by only 2.2 points between 4 and 5, and in the quadratic model it rose by 2.6 points between perceived stress scores of 4 and 6 but was flat above about 10. Both predictors remained strongly associated with food noise when linear and non-linear terms were tested together (perceived stress F = 11.93, *p* < 0.001; SCOFF F = 62.91, *p* < 0.001). The two weaker associations in the primary model did not hold: age was no longer significant when tested jointly with its quadratic term (F = 2.50, *p* = 0.083), and any night shift work fell to B 1.76 (*p* = 0.117) in the quadratic model and B 1.87 (*p* = 0.097) in the spline model. Allowing non-linearity also reduced but did not remove the evidence of misspecification, with the Ramsey RESET test moving from *p* < 0.001 in the primary model to *p* = 0.053 with quadratic terms and *p* = 0.036 with splines.

Taken together, the six analyses separate the findings into two groups. The associations with eating disorder risk and perceived stress held in every specification and are robust, although their shape is concave rather than linear. The associations with age and with night shift work did not: age failed once non-linearity was allowed, and night shift work failed under the original categorical coding, under both non-linear specifications, and after the exclusion of influential observations, holding only in the primary specification and the missing category variant. We therefore treat the first two as established within the limits of a cross-sectional convenience sample and the second two as provisional.

## 4. Discussion

In this cross-sectional study of 593 healthcare students and professionals in Al-Ahsa, food noise was common, and its severity varied widely. Three quarters of participants met the study-defined threshold for at least moderate food noise, and close to a third fell into the high-severity band. The 14-item scale proved highly reliable and unidimensional. Once the predictors were adjusted for one another, food noise remained independently associated with eating disorder risk and perceived stress, and these two associations held in every sensitivity analysis; weaker associations with age and night shift work appeared in the primary model but did not survive alternative specifications, and the crude differences by sex, role, weekly hours, and fasting did not hold up at all. To our knowledge, this is the first study to quantify food noise in a healthcare population in Saudi Arabia.

The high proportion of participants meeting the study-defined threshold fits the idea that food noise reflects food cue reactivity operating along a continuum, from an ordinary feature of appetite to a more intrusive, distressing experience [1,4]. Because the construct captures normal thoughts about food as well as problematic ones, some degree of it is expected in any general sample, and we chose mid-point thresholds precisely to separate this background level from more intrusive experience. The mean item scores clustered around the neutral point, with involuntary and recurrent thoughts rated more highly than deliberate avoidance patterns, which fits the cognitive account in which intrusions are easier to register than to suppress [5,6].

The strongest correlate of food noise was eating disorder risk on the SCOFF screen, which stayed the dominant predictor in the adjusted model. That fits conceptual models placing food cue reactivity, craving, and impaired cognitive control at the heart of disordered eating, on which intrusive food thoughts and disordered eating attitudes would naturally travel together [5,18]. Part of the association is likely to reflect shared item content rather than a relationship between two independent constructs, as SCOFF item 5 (“Would you say food dominates your life?”) is close in meaning to food noise items 3 and 14. We tested this directly. Removing the two overlapping food noise items did not weaken the association at all, and their item-level correlations with SCOFF item 5 were among the lowest in the scale rather than the highest; removing SCOFF item 5 reduced the correlation from 0.48 to 0.40, and the adjusted association remained strong when both instruments were reduced. Content redundancy therefore inflates the estimate modestly but does not generate it, and the association should still be read as one between two overlapping self-report measures rather than as validation of food noise against an independent criterion.

The proportion screening positive on the SCOFF, 70.2%, is high enough to require explanation rather than a caveat. It sits well above the range reported for Saudi adolescents and young adults, where a systematic review of 14 studies found disordered eating in 10.2% to 48.1% overall, with the highest figures, 29.4% to 65.5%, in the Eastern Province, the region in which Al-Ahsa lies [32]. It is also above the figures reported in comparable student samples using the same or similar instruments [17,25]. Three explanations deserve weight. The first is measurement. The SCOFF has a pooled sensitivity of 0.86 and specificity of 0.83 in general-population validation studies [33] and 0.84 and 0.80 in the evidence review prepared for the US Preventive Services Task Force [34]. Applying a Rogan–Gladen correction for these test characteristics does not lower the estimate; it raises it, to approximately 77%, because imperfect specificity accounts for only about 4 percentage points of the observed total. Imprecision of the instrument alone therefore cannot explain the figure. The second explanation is item interpretation in the translated version. Endorsement of the first SCOFF item, which asks about deliberate vomiting when uncomfortably full, reached 43.2%, an implausible rate of self-induced vomiting in a non-clinical population and one that points to respondents reading the item as asking about nausea or the urge to vomit rather than about a purging behaviour. Endorsement of 34.2% for the loss of more than 6.5 kg in three months is similarly high. Both readings would inflate the total score without reflecting the intended construct. The third is self-selection, discussed below. Taken with the absence of any diagnostic confirmation, these considerations mean the SCOFF figure should be read as a screen-positive proportion in a self-selected sample and not as an estimate of eating disorder prevalence in Al-Ahsa.

Perceived stress was the second independent correlate. This sits well with evidence that psychological stress engages the hypothalamic–pituitary–adrenal axis and reward pathways and can sharpen the salience of food and with student data linking higher stress to altered eating [10,11]. In healthcare training, where stress and burnout are well documented, a stress-related amplification of food thoughts is a reasonable reading—though, with a cross-sectional design, we cannot establish which way the relationship runs [8,9].

Older age went with slightly greater food noise in the primary model. Since professionals were, on average, older than students (30.3 against 22.7 years), age and role were partly entangled, although not to a degree that destabilised the model (variance inflation factor 1.63 for role); the crude student–professional gap disappeared after adjustment, which suggests that age, stress, and eating disorder risk accounted for much of it. The age association is not robust, however, and disappears once the continuous predictors are allowed to depart from linearity, so we do not regard it as established. Night shift work is in the same position. The crude gradient was steep, and the association survived adjustment in the primary specification, which is biologically plausible given that sleep loss and circadian misalignment act on appetite-regulating hormones and on weight [12,13,15], but it did not survive the original categorical coding of the variable, either non-linear specification, or the exclusion of influential observations. The most that can be said is that participants who work any night shifts scored higher than those who work none, by about two to six points depending on the model, and that the data cannot distinguish this from chance once the model is specified differently. The bivariate pattern does at least argue against a dose–response effect: participants working one, two, or three or more shifts per month scored within 1.6 points of one another, and their adjusted coefficients did not differ (*p* = 0.921), so what distinguishes the groups is exposure to night work rather than its frequency. Confirmation in a study designed around shift exposure is needed before the relationship is treated as real.

Whether food noise behaves this way outside healthcare settings, and outside this cultural context, is an open question. The construct has so far been described mainly in North American and European samples, largely in the context of obesity treatment and glucagon-like peptide-1 receptor agonist use [1,2,3], and the mechanisms invoked to explain it, cue reactivity and the elaboration of craving in working memory, are not specific to any occupation. Healthcare students and professionals were chosen here because the conditions thought to amplify those mechanisms, sustained stress, irregular hours, and disrupted meal timing, are concentrated in this group and are documented in it [8,9,15,16] and because they are also the people who will be asked to recognise and counsel patients about intrusive food thoughts. Other occupations with the same exposures, such as shift-working industrial and transport staff, would be informative comparison groups, as would non-shift office populations. The wider point is that the two variables that mattered here, stress and eating disorder risk, are not occupation-specific, so we would expect the construct to appear in any high-stress population; testing that expectation requires studies in occupations outside healthcare and in countries with different eating cultures.

Religious fasting is one feature of this context that deserves comment even though it dropped out of the adjusted model. Sixty-one per cent of participants reported fasting regularly, and in unadjusted comparison they scored three points higher on the food noise scale (43.1 against 40.1, *p* = 0.009), a difference that did not survive adjustment for stress and eating disorder risk. Voluntary fasting outside Ramadan is common in Saudi Arabia and involves long daylight abstinence followed by concentrated evening eating, a pattern that would plausibly increase the frequency and salience of food thoughts during the fasting hours. Two features of the present design limit what can be said. The questionnaire asked only whether the respondent fasted regularly, without recording the type of fast, its frequency, or whether data collection coincided with Ramadan, and the scale asks about intrusive food thoughts without reference to a time frame, so a respondent fasting on the day of completion and one who fasts twice a month would answer the same question differently. The attenuation after adjustment should therefore not be read as evidence that fasting is unimportant, but as an indication that a purpose-built study, ideally comparing the same individuals inside and outside a fasting period, is needed to separate the cultural practice from the psychological state that accompanies it.

The findings carry practical weight. Food noise has become a familiar shorthand for patients and clinicians describing appetite, partly through experience with glucagon-like peptide-1 receptor agonists, and a simple self-report measure may help flag those whose eating is shaped by intrusive food thoughts [2,3]. In a setting where obesity and poor dietary patterns are common, paying attention to the psychological side of eating among current and future health workers could inform wellbeing and nutrition programmes in training institutions and hospitals [23,24]. Three implications follow from the pattern observed here, with each of them being provisional. First, because food noise tracked eating disorder risk and perceived stress rather than role or workload, wellbeing services in health professions education would be better placed to address it through existing stress and disordered eating pathways than through occupational scheduling policy. Second, the strength of the association with the SCOFF screen suggests that students and staff who describe persistent intrusive thoughts about food are a group in whom a brief eating disorder screen is worth offering, given that a positive screen was three times more common in the high-severity band than in the low. Third, the night shift finding, if confirmed, would argue for attention to meal availability and timing on night rotas, which is a modifiable feature of hospital catering rather than of the rota itself. None of these follows directly from a cross-sectional study, and each would need testing before being adopted.

The study has several strengths. The sample was large and drawn from a range of healthcare disciplines; eating disorder risk and perceived stress were measured with established instruments; and the food noise scale showed high internal consistency with uniformly positive item-rest correlations. Operational definitions were transparent and prespecified, and we retained the analytic dataset for reproducibility.

Several limitations temper these findings. The cross-sectional design rules out causal inference: the associations described here are statistical, and reverse or bidirectional links between stress, disordered eating, and food noise are as compatible with the data as the direction in which we have presented them. Recruiting by convenience through electronic channels is the most serious constraint. Distribution through social media and a survey-distribution service invites participation from people already interested in nutrition, dieting, or weight, and no denominator exists from which a response rate could be calculated, so the proportions reported here are almost certainly higher than they would be in a random sample of healthcare students and professionals in the governorate; the associations between variables are less vulnerable to this than the proportions, but they are not immune, since a sample enriched for food-related concern will compress the range of the outcome. Distribution through institutional and social media channels also means that respondents from the same class or department are likely to be over-represented, so observations may not be fully independent, and no clustering variable was recorded that would allow this to be modelled. Every measure was self-reported and therefore open to recall and social-desirability effects. No validated diagnostic cut-off for food noise yet exists, so our severity categories, though prespecified, stay provisional and are not directly comparable across studies [1]. The food noise scale itself was adapted from an instrument built to measure thought suppression rather than food noise, so its construct validity for the target concept rests on conceptual argument and internal structure rather than on evidence against an external criterion, and the Arabic version has not been formally validated. The psychometric analyses were carried out in the same sample that generated the scale, and although the structure replicated across random halves, that is not equivalent to confirmation in an independent sample. The SCOFF screen flags probable risk rather than diagnosed eating disorders, tends to over-identify in non-clinical groups [19], and shares item content with the food noise scale. Body mass index could not be calculated because no height or weight values were available in the analysis dataset, and we did not capture current dieting, previous eating disorder diagnosis, psychotropic or glucagon-like peptide-1 receptor agonist use, mood, or metabolic conditions. Body weight status is plausibly related both to food-related cognition and to eating disorder risk, so its absence is the single most likely source of residual confounding in the adjusted model, and the coefficients for eating disorder risk and perceived stress may be inflated to the extent that both travel with body weight. No a priori sample size calculation was performed. The collapsing of physical activity and night shift frequency into binary variables was informed by the observed data rather than prespecified, and although a sensitivity analysis retaining the original categories is reported, this is a departure from a fully prespecified analysis plan. Regression assumptions were checked rather than assumed, and the tests indicated heteroscedasticity and departure from linearity; robust standard errors address the first, and models allowing quadratic and spline terms address most but not all of the second, so the per-point coefficients in Table 5 should be read as average effects across the observed range rather than as constant increments. The four-item stress measure, while practical, is also the least precise of the Perceived Stress Scale forms [22]. Longitudinal, multicentre studies using probability sampling, anthropometric measurement, and anchored or validated food noise measures would help confirm these associations and clarify their direction.

## 5. Conclusions

Food noise was common among the healthcare students and professionals who took part in this study in Al-Ahsa, and it spanned a wide range of severity, with three quarters of participants meeting the study-defined threshold for at least moderate intrusion. Its intensity tracked eating disorder risk and perceived stress, and these two associations held across every sensitivity analysis, although both relationships are concave rather than linear, so the increment attached to each additional point falls as scores rise. Weaker associations with age and with night shift work did not survive alternative specifications and should be treated as provisional. Demographic and occupational characteristics otherwise explained little. The 14-item scale was internally consistent and unidimensional in this sample, which supports its provisional use while falling short of establishing its validity. Because the sample was recruited by convenience and the severity thresholds were defined from the response scale rather than from any clinical criterion, the proportions reported here describe this sample rather than the population of Al-Ahsa. These results offer a first benchmark for the construct in this population and point to the cognitive side of eating as a target for wellbeing and nutrition efforts in health profession training and clinical care. What is needed now are longitudinal, multicentre studies with probability sampling and formally validated food noise measures to confirm these associations and clarify their direction.

## Figures and Tables

**Table 1:** Demographic and lifestyle characteristics of the study sample (*n* = 593).

Characteristic	*n* (%)
Age, years, mean (SD)	25.7 (6.6)
Sex	
Female	400 (67.5)
Male	193 (32.5)
Role group	
Student	356 (60)
Professional	237 (40)
Discipline	
Nursing	202 (34.1)
Pharmacy	195 (32.9)
Medicine	148 (25)
Other allied health fields	48 (8.0)
Weekly work or study hours	
Less than 40 h/week	152 (25.6)
40 h/week	255 (43)
More than 40 h/week	173 (29.2)
Physical activity	
None	239 (40.3)
Irregular	200 (33.7)
Regular	153 (25.8)
Night shifts per month	
None	275 (46.4)
One	79 (13.3)
Two	112 (18.9)
Three or more	111 (18.7)
Regular fasting, yes	361 (60.9)

Note. Percentages are based on the full sample of 593. Values that could not be assigned to a category were as follows: weekly work or study hours (13), physical activity (1), and night shifts per month (16). Most of these were free-text answers that did not correspond to any response option rather than skipped items. SD, standard deviation.

**Table 2:** Item-level descriptive statistics, item-rest correlations, and exploratory factor loadings for the 14-item food noise scale (*n* = 593).

Item	Mean	SD	ItemRest r	Factor Loading
1. I deliberately try not to think about food	2.84	1.28	0.47	0.50
2. Thoughts about food come to me against my will	3.21	1.27	0.53	0.56
3. I think about food constantly	3.07	1.24	0.60	0.63
4. I wish I could stop thinking about food	3.09	1.36	0.62	0.65
5. There are images of food I cannot erase from my mind	2.95	1.40	0.57	0.60
6. I do my best to suppress my thoughts about food	2.94	1.38	0.68	0.71
7. Food thoughts stop me from concentrating on other things	2.97	1.41	0.62	0.66
8. I have thoughts about food that keep coming back again and again	3.02	1.32	0.66	0.70
9. I often think about food when I should not	2.97	1.34	0.66	0.70
10. I try hard to push food thoughts out of my mind	2.99	1.40	0.70	0.74
11. I have thoughts about food I would rather not think about	2.88	1.35	0.62	0.65
12. I avoid thinking about food	3.03	1.39	0.64	0.67
13. Food thoughts interrupt me during my daily activities	3.02	1.35	0.60	0.64
14. I have many thoughts about food every day	2.95	1.30	0.59	0.62
Total food noise score (14–70)	41.9	12.7	–	–

Note. Each item scored 1 (strongly disagree) to 5 (strongly agree). Item-rest r is the correlation between the item and the sum of the remaining 13 items. Factor loadings are from a one-factor principal-axis solution (Kaiser–Meyer–Olkin 0.95; Bartlett χ^2^ = 3301.9, *df* = 91, *p* < 0.001; 41.8% of variance explained). SD, standard deviation. The Cronbach alpha for the full scale was 0.909.

**Table 3:** SCOFF item endorsement and screen result (*n* = 593).

SCOFF Item (Affirmative Response)	*n*	%
Sick (vomit when full)	256	43.2
Control (lost control overeating)	291	49.1
One stone (>6.5 kg/3 mo)	203	34.2
Fat (feel fat when thin)	257	43.3
Food (food dominates life)	272	45.9
Positive SCOFF screen (score ≥ 2)	416	70.2

Note. Each affirmative response contributes one point to the SCOFF total (range 0 to 5).

**Table 4:** Bivariate associations between participant characteristics and the food noise total score, with false discovery rate correction.

Characteristic	Group 1/Detail	Group 2	Test	*p*	q
Gender	Male (*n* = 193): 43.6 ± 11.1	Female (*n* = 400): 41.1 ± 13.4	t = 2.37, d = 0.19	0.018	0.026
Role group	Student (*n* = 356): 41.1 ± 13.4	Professional (*n* = 237): 43.2 ± 11.5	t = −1.99, d = −0.16	0.047	0.059
Fasting	Yes (*n* = 361): 43.1 ± 10.9	No (*n* = 232): 40.1 ± 14.9	t = 2.63, d = 0.24	0.009	0.015
SCOFF screen	Positive (*n* = 416): 45.6 ± 10.9	Negative (*n* = 177): 33.3 ± 12.5	t = 11.40, d = 1.08	<0.001	<0.001
Weekly work/study hours	<40 h/week (*n* = 152): 40.7 ± 12.2; 40 h/week (*n* = 255): 42.1 ± 12.3; >40 h/week (*n* = 173): 43.2 ± 13.5		F = 1.59, η^2^ = 0.005	0.206	0.229
Physical activity	None (n = 239): 41.8 ± 13.2; Irregular (*n* = 200): 42.3 ± 12.1; Regular (*n* = 153): 41.6 ± 12.9		F = 0.16, η^2^ = 0.001	0.856	0.856
Night shifts/month	None (*n* = 275): 38.7 ± 15.2; 1 (*n* = 79): 44.8 ± 9.0; 2 (*n* = 112): 45.6 ± 7.9; 3+ (*n* = 111): 44.0 ± 10.3		F = 12.14, η^2^ = 0.060	<0.001	<0.001
Age (years)	*n* = 593; Pearson correlation with food noise total		r = 0.12	0.004	0.008
Perceived stress (PSS-4)	*n* = 593; Pearson correlation with food noise total		r = 0.22	<0.001	<0.001
SCOFF total score	*n* = 593; Pearson correlation with food noise total		r = 0.48	<0.001	<0.001

Note. Values for two-group comparisons are mean ± standard deviation. For the three continuous characteristics the group columns do not apply and are merged. t, independent-samples *t*-test; F, one-way analysis of variance; r, Pearson correlation; d, Cohen d; η^2^, eta-squared. q, Benjamini–Hochberg false discovery rate across the ten tests in this table. Group sizes for the analysis of variance rows exclude participants with a missing value on that variable.

**Table 5:** Crude and adjusted linear regression for the food noise total score (complete-case *n* = 573).

Predictor	Crude B (95% CI)	Adjusted B (95% CI)	β	VIF	*p*	p (HC3)
Age (per year)	0.22 (0.07 to 0.38)	0.19 (0.02 to 0.36)	0.10	1.52	0.026	0.008
Male (vs. female)	2.37 (0.20 to 4.55)	0.38 (−1.61 to 2.37)	0.01	1.09	0.705	0.683
Professional (vs. student)	2.10 (0.02 to 4.18)	−0.34 (−2.66 to 1.99)	−0.01	1.63	0.777	0.751
Regular physical activity (vs. not)	−0.55 (−2.88 to 1.79)	−0.92 (−3.00 to 1.16)	−0.03	1.05	0.387	0.418
Any night shifts (vs. none)	6.10 (4.07 to 8.13)	2.45 (0.29 to 4.60)	0.10	1.45	0.026	0.024
>40 h/week (vs. 40 h)	1.77 (−0.45 to 3.99)	−0.20 (−2.37 to 1.97)	−0.01	1.24	0.856	0.858
<40 h/week (vs. 40 h)	−2.31 (−4.61 to −0.01)	−1.87 (−4.11 to 0.36)	−0.07	1.22	0.101	0.099
Regular fasting (vs. not)	2.87 (0.79 to 4.95)	1.30 (−0.65 to 3.25)	0.05	1.12	0.192	0.208
Perceived stress (per point)	0.98 (0.62 to 1.33)	0.69 (0.36 to 1.02)	0.15	1.09	<0.001	<0.001
SCOFF score (per point)	4.38 (3.73 to 5.03)	3.85 (3.15 to 4.55)	0.42	1.16	<0.001	<0.001

Note. B, unstandardised coefficient; CI, confidence interval; β, standardised coefficient; VIF, variance inflation factor. Crude coefficients come from single-predictor models fitted on all participants with a value for that predictor; adjusted coefficients come from the complete-case multivariable model (*n* = 573). Reference categories: female sex, student role, no night shifts, and 40 h per week. *p* (HC3) is the *p* value obtained with heteroscedasticity-consistent standard errors. Model R-squared = 0.279, adjusted R-squared = 0.266.

**Table 6:** Sensitivity analyses for the association between food noise and its principal correlates.

Analysis	*n*	SCOFF	Stress	Age	Night Shifts	R^2^
Primary model (complete case)	573	3.85 (3.15 to 4.55)	0.69 (0.36 to 1.02)	0.19 (0.02 to 0.36)	2.45 (0.29 to 4.60)	0.279
All participants, missing category indicator	593	3.88 (3.20 to 4.57)	0.64 (0.32 to 0.97)	0.19 (0.03 to 0.35)	2.37 (0.24 to 4.50)	0.284
Excluding Cook’s distance > 4/*n*	546	4.16 (3.54 to 4.79)	1.02 (0.72 to 1.32)	0.21 (0.06 to 0.36)	1.51 (−0.40 to 3.41)	0.386
Reduced scales (12-item food noise, 4-item SCOFF)	573	3.42 (2.66 to 4.18)	0.66 (0.36 to 0.96)	0.18 (0.03 to 0.33)	–	0.239
Original categorical coding of activity and shifts	573	3.85 (3.15 to 4.55)	0.69 (0.36 to 1.02)	0.19 (0.02 to 0.36)	joint *p* = 0.165	0.279
Quadratic terms for age, stress and SCOFF	573	joint *p* < 0.001	joint *p* < 0.001	joint *p* = 0.083	1.76 (−0.44 to 3.96)	0.300
Restricted cubic splines for age, stress and SCOFF	573	joint *p* < 0.001	joint *p* = 0.002	joint *p* = 0.265	1.87 (−0.34 to 4.07)	0.302

Note. Values are unstandardised coefficients with 95% confidence intervals unless stated otherwise. All models are adjusted for age, sex, role group, physical activity, night shift work, weekly hours, regular fasting, perceived stress, and the SCOFF score. In the two non-linear models the effect of a continuous predictor is not a single per-point coefficient, so the joint test of its linear and non-linear terms is given instead. The reduced-scale model omits food noise items 3 and 14 and SCOFF item 5 and so is not directly comparable in scale with the others. The first four analyses were prespecified; the last two were added in revision.

## Data Availability

All data are available upon request from the corresponding author.

## Supplementary Materials

The following supporting information can be downloaded at: https://www.mdpi.com/article/10.3390/nu18172824/s1. Supplementary File S1: Study questionnaire (demographic and lifestyle items, the 14-item food noise scale, the SCOFF screen, the four-item Perceived Stress Scale, and the musculoskeletal item), in both the Arabic and English versions administered; Supplementary Figure S1: Scree plot of the food noise scale with the parallel analysis threshold.

## Appendix A. STROBE Statement—Checklist for Cross-Sectional Studies

The following checklist indicates where each STROBE item is addressed in the manuscript. For measurement items, the corresponding questionnaire sections and item numbers are noted.

**No.**	**Recommendation**	**Reported in/Questionnaire Items**
1	Title and abstract: (a) study design in title or abstract; (b) informative and balanced abstract	Title; Abstract
2	Background/rationale: scientific background and rationale	Introduction (paragraphs 1–4)
3	Objectives: specific objectives/hypotheses	Introduction (final paragraph)
4	Study design: present key elements early in the paper	Methods—Study design and setting
5	Setting: locations, relevant dates, recruitment, data collection	Methods—Study design and setting
6	Participants: eligibility criteria, sources, selection methods	Methods—Participants and eligibility
7	Variables: outcomes, exposures, predictors, confounders	Methods—Questionnaire and measures. Q items: Section S1 (demographics/lifestyle, incl. fasting, night shifts); Section S2 food noise (14 items as administered); Section S3 SCOFF (items 1–5); Section S4 PSS-4 (items 1–4); and a single musculoskeletal pain item based on the Nordic questionnaire
8	Data sources/measurement: methods of assessment for each variable	Methods—Questionnaire and measures; Operational definitions (same questionnaire items as item 7)
9	Bias: efforts to address potential sources of bias	Methods (convenience sampling); Discussion—Limitations
10	Study size: how the study size was arrived at	Results—Participant flow (*n* = 593); Methods—Statistical analysis (precision of the achieved sample; no a priori calculation)
11	Quantitative variables: handling of quantitative variables/groupings	Methods—Operational definitions; Statistical analysis
12	Statistical methods: all methods, subgroups, missing data, sensitivity	Methods—Statistical analysis (incl. EFA: KMO, Bartlett, principal-axis factoring, parallel analysis, split-half replication; complete-case handling of missing data; assumption checks; false discovery rate; sensitivity analyses)
13	Participants: numbers at each stage, reasons for non-participation	Results—Participant flow and sample characteristics
14	Descriptive data: characteristics of participants; missing data	Results—Table 1 and note (missing values by variable)
15	Outcome data: numbers/summary measures of outcomes	Results—Table 2 and Table 3; Figure 1 and Figure 2
16	Main results: estimates and precision (e.g., 95% CI); confounder adjustment	Results—Table 4 and Table 5; Multivariable analysis
17	Other analyses: subgroups, interactions, sensitivity analyses	Results—Factors associated with food noise; Multivariable analysis; Sensitivity analyses (Table 6)
18	Key results: summarise with reference to study objectives	Discussion (first paragraph)
19	Limitations: sources of bias/imprecision and direction	Discussion—Limitations
20	Interpretation: cautious overall interpretation	Discussion; Conclusions
21	Generalisability: external validity	Discussion—Limitations
22	Funding: role of funders	Funding statement
