# Supplementary material for: Food Noise Among Healthcare Students and Healthcare Professionals in Al-Ahsa, Saudi Arabia: A Cross-Sectional Study
Source: Nutrients. 2026 Aug 28;18(17):2824. doi: 10.3390/nu18172824 (PMC13567203; doi:10.3390/nu18172824)
Supplement: Supplementary file 1 [file nutrients-18-02824-s001.zip › nutrients-4439041-Supplementary File S1.pdf]

Supplementary File S1

Study Questionnaire as Administered

Food Noise among Healthcare Students and Healthcare Professionals in Al-Ahsa, Saudi Arabia: A Cross-Sectional Study

This file reproduces the questionnaire in the form in which it was administered. The Arabic column gives the item wording exactly as it appeared in the deployed electronic form; the English column gives the corresponding English wording. Response options are listed under each item in the order in which they were presented. Items were single-select unless otherwise stated.

Consent

You are invited to participate in a research study about thoughts and experiences related to food, stress, and health. Participation is voluntary, anonymous, and takes about 8 to 10 minutes. You may skip any question or stop at any time. No names or personal identifiers are collected. Your answers will help improve wellbeing programmes for healthcare students and professionals.

English	العربية
Do you consent to participate in this study?	هل توافق على المشاركة في هذه الدراسة؟
Yes, I consent to participate. / No, I do not consent (exit the survey).	نعم / لا

Section S1. Demographic and lifestyle characteristics (7 items)

English	العربية
1. Age (years). Free text.	العمر (بالسنوات)
2. Sex. Options: Male; Female.	الجنس — ذكر / أنثى
3. Role. Free text; responses were subsequently classified into a healthcare discipline and into a student or professional category using a predefined scheme.	الدور — نص حر
4. Average weekly work or study hours. Options: fewer than 8 hours daily (equivalent to fewer than 40 hours weekly); 8 hours daily (equivalent to 40 hours weekly); more than 8 hours daily (equivalent to more than 40 hours weekly).	متوسط عدد ساعات العمل/الدراسة أسبوعيًا — أقل من 8 ساعات يوميًا (بما يعادل أقل من 40 ساعة أسبوعيًا) / 8 ساعات يوميًا (بما يعادل 40 ساعة أسبوعيًا) / أكثر من 8 ساعات يوميًا (بما يعادل أكثر من 40 ساعة أسبوعيًا)
5. Do you exercise more than 3 times per week, averaging 150 minutes? Options: I do not exercise; I exercise irregularly; Yes, I exercise regularly.	هل تمارس الرياضة أكثر من 3 مرات أسبوعيًا بمعدل 150 دقيقة — لا أمارس الرياضة / أمارس الرياضة بشكل غير منتظم / نعم، أمارس الرياضة بانتظام
6. How many night shifts do you work per month (if any)? Options: none; one shift; two shifts; three shifts. This item could be left unanswered.	كم يبلغ عدد المناوبات الليلية التي تؤديها في الشهر (إن وجدت)؟ — لا يوجد / مناوبة واحدة / مناوبتان / 3 مناوبات
7. Do you fast regularly, for religious or health reasons? Options: Yes; No.	هل تصوم بانتظام (لأسباب دينية أو صحية) — نعم / لا

No anthropometric items were included: height and weight were not collected, and body mass index could not therefore be derived.

Section S2. Food noise scale (14 items)

Adapted from the Food Thought Suppression Inventory. Each item was rated on a five-point scale: 1 = strongly disagree, 2 = disagree, 3 = neutral, 4 = agree, 5 = strongly agree.

١ = أعارض بشدة، ٢ = أعارض، ٣ = محايد، ٤ = أوافق، ٥ = أوافق بشدة

**Item**	**البند**
1. I deliberately try not to think about food.	أحاول عمدًا ألا أفكر بالطعام
2. Thoughts about food come to me against my will.	تراودني أفكار عن الطعام رغمًا عني
3. I think about food constantly.	أفكر باستمرار في الطعام
4. I wish I could stop thinking about food.	أتمنى أن أتوقف عن التفكير في الطعام
5. There are images of food I cannot erase from my mind.	هناك صور عن الطعام لا أستطيع محوها من ذهني
6. I do my best to suppress my thoughts about food.	أبذل قصارى جهدي لكبت أفكاري عن الطعام
7. Food thoughts stop me from concentrating on other things.	تمنعني أفكار الطعام من التركيز على أشياء أخرى
8. I have thoughts about food that keep coming back again and again.	لدي أفكار عن الطعام تعود مرارًا وتكرارًا
9. I often think about food when I should not.	كثيرًا ما أفكر في الطعام عندما لا ينبغي لي ذلك
10. I try hard to push food thoughts out of my mind.	أحاول جاهدًا طرد أفكار الطعام من ذهني
11. I have thoughts about food I would rather not think about.	لدي أفكار عن الطعام أفضّل أن لا أفكر بها
12. I avoid thinking about food.	أتجنب التفكير في الطعام
13. Food thoughts interrupt me during my daily activities.	تقاطعني أفكار الطعام أثناء أنشطتي اليومية
14. I have many thoughts about food every day.	لدي العديد من الأفكار عن الطعام كل يوم

The original Food Thought Suppression Inventory comprises 15 items. The item "I have thoughts about food I cannot stop" was judged to overlap conceptually with item 3 above and was omitted when the questionnaire was built, so the instrument administered to participants contained the 14 items listed here.

Section S3. SCOFF eating-disorder screen (5 items)

Each item was answered Yes or No. One point was assigned for each affirmative answer, giving a total of 0 to 5.

**Item**	**البند**
1. Do you make yourself sick because you feel uncomfortably full?	هل تتقيأ عمدًا لأنك تشعر بالامتلاء المفرط؟
2. Do you worry you have lost control over how much you eat?	هل تقلق من أنك فقدت السيطرة على كمية الطعام التي تتناولها؟
3. Have you recently lost more than 6.5 kg in a 3-month period?	هل فقدت مؤخرًا أكثر من 6.5 كجم خلال 3 أشهر؟
4. Do you believe yourself to be fat when others say you are too thin?	هل تعتقد أنك بدين بينما يقول الآخرون أنك نحيف جدًا؟

Item	البند
5. Would you say food dominates your life?	هل يمكن القول أن الطعام يهيمن على حياتك؟

Section S4. Perceived Stress Scale, four-item version (4 items)

Each item was rated: 0 = never, 1 = almost never, 2 = sometimes, 3 = fairly often, 4 = very often. Items 3 and 4 were reverse-scored.

٠ = أبدًا، ١ = نادرًا جدًا، ٢ = نادرًا، ٣ = غالبًا، ٤ = دائمًا

Item	البند
1. In the last month, how often have you felt that difficulties were piling up so high that you could not overcome them?	خلال الشهر الماضي، كم مرة شعرت أن الصعوبات تتراكم بحيث لا تستطيع التغلب عليها؟
2. In the last month, how often have you felt unable to control the important things in your life?	خلال الشهر الماضي، كم مرة شعرت أنك غير قادر على التحكم في الأمور المهمة في حياتك؟
3. In the last month, how often have you felt confident about your ability to handle your personal problems? (reverse-scored)	خلال الشهر الماضي، كم مرة شعرت بالثقة في قدرتك على التعامل مع مشاكلك الشخصية؟ (يُعكس التقييم)
4. In the last month, how often have you felt that things were going your way? (reverse-scored)	خلال الشهر الماضي، كم مرة شعرت أن الأمور تسير كما ترغب؟ (يُعكس التقييم)

Section S5. Musculoskeletal symptoms (1 item)

Based on the Standardised Nordic Musculoskeletal Questionnaire and its validated Arabic adaptation. A single item, with one option selected.

Item	البند
In the past 12 months, have you had pain or discomfort in any of the following areas that affected your work or daily activities? Options: neck; shoulders; lower back; knees; no pain or discomfort.	خلال 12 شهرًا الماضية، هل عانيت من ألم أو إزعاج في المناطق التالية أثر على عملك أو أنشطتك اليومية؟ — الرقبة / الكتفين / أسفل الظهر / الركبتين / لا يوجد ألم أو إزعاج

Closing text

Thank you for your participation in this study. If you experience any distress related to food, body image, or stress after completing this survey, we encourage you to seek appropriate professional support. In Saudi Arabia, you may contact the Ministry of Health service line (937) or your university's counselling service.

Instrument references

1. Barnes, R.D.; Masheb, R.M.; White, M.A.; Grilo, C.M. Validation of the Food Thought Suppression Inventory. *J. Health Psychol.* **2010**, *15*, 373–381. https://doi.org/10.1177/1359105309351246.

2. Morgan JF, Reid F, Lacey JH. The SCOFF questionnaire: assessment of a new screening tool for eating disorders. BMJ. 1999;319(7223):1467-1468.

3. Cohen S, Kamarck T, Mermelstein R. A global measure of perceived stress. J Health Soc Behav. 1983;24(4):385-396.

4. Kuorinka I, Jonsson B, Kilbom A, Vinterberg H, Biering-Sorensen F, Andersson G, Jorgensen K. Standardised Nordic questionnaires for the analysis of musculoskeletal symptoms. Appl Ergon. 1987;18(3):233-237.
